# White Matter Changes-Related Gait and Executive Function Deficits: Associations with Age and Parkinson's Disease

**DOI:** 10.3389/fnagi.2017.00213

**Published:** 2017-06-30

**Authors:** Jennifer Sartor, Kristina Bettecken, Felix P. Bernhard, Marc Hofmann, Till Gladow, Tobias Lindig, Meltem Ciliz, Mara ten Kate, Johanna Geritz, Sebastian Heinzel, Marije Benedictus, Philip Scheltens, Markus A. Hobert, Walter Maetzler

**Affiliations:** ^1^Department of Neurodegeneration, Center for Neurology and Hertie Institute for Clinical Brain Research, University of Tuebingen Tuebingen, Germany; ^2^German Center for Neurodegenerative Diseases (DZNE) Tuebingen, Germany; ^3^Hasomed GmbH Magdeburg, Germany; ^4^Department of Diagnostic and Interventional Neuroradiology, University of Tuebingen Tuebingen, Germany; ^5^Alzheimer Center and Department of Neurology, Neuroscience Campus Amsterdam, VU University Medical Center Amsterdam, Netherlands; ^6^Department of Neurology, Christian-Albrechts-University of Kiel Kiel, Germany

**Keywords:** cognition, dual tasking, older adults, Parkinson's disease, white matter changes

## Abstract

**Background:** White matter changes (WMC) are a common finding among older adults and patients with Parkinson's disease (PD), and have been associated with, e.g., gait deficits and executive dysfunction. How the factors age and PD influence WMC-related deficits is, to our best knowledge, not investigated to date. We hypothesized that advanced age and presence of PD leads to WMC-related symptoms while practicing tasks with a low complexity level, and low age and absence of PD leads to WMC-related symptoms while practicing tasks with a high complexity level.

**Methods:** Hundred and thirty-eight participants [65 young persons without PD (50–69 years, yPn), 22 young PD patients (50–69 years, yPD), 36 old persons without PD (70–89 years, oPn) and 15 old PD patients (70–89 years, oPD)] were included. Presence and severity of WMC were determined with the modified Fazekas score. Velocity of walking under single and dual tasking conditions and the Trail Making Test (TMT) were used as gait and executive function parameters. Correlations between presence and severity of WMC, and gait and executive function parameters were tested in yPn, yPD, oPn, and oPD using Spearman's rank correlation, and significance between groups was evaluated with Fisher's z-transformed correlation coefficient.

**Results:** yPn and yPD, as well as oPn and oPD did not differ regarding demographic and clinical parameters. Severity of WMC was not significantly different between groups. yPn and yPD displayed significant correlations of WMC with executive function parameters at low levels of task complexity, oPn at intermediate, and oPD at high complexity levels.

**Conclusion:** This study argues for a relevant association of age and PD-related brain pathology with WMC-related gait and executive function deficits.

## Introduction

White matter changes (WMC) are a common finding in brain imaging such as MRI. They increase with age (Breteler et al., 1994) and are thought to reflect microangiopathy (Schmidt et al., 2004). Evidence emerges that WMC have a relevant influence on daily activity- and life quality-relevant features such as gait and executive functioning. This has been shown for older adults in general (Marshall et al., 2006; Baezner et al., 2008; de Laat et al., 2010; Smith et al., 2011; Zheng et al., 2012) and for patients with Parkinson's disease (PD) (Sohn and Kim, 1998; Kandiah et al., 2013; Theilmann et al., 2013; Veselý et al., 2015; Veselý and Rektor, 2016) in particular. The influence on both, gait and executive functioning, is plausible as a dynamically expanding mass of scientific literature shows that gait functioning depends on the input from supraspinal centers, especially those associated with executive functioning (meaning mental faculties of controlled, planned and anticipative behavior) (Grillner, 2003; Jahn et al., 2004; Grillner and Jessell, 2009; Maetzler et al., 2013).

The occurrence and frequency of falls, lowered gait velocity as well as gait and balance disorders are significantly correlated with WMC in higher age (Baezner et al., 2008; Blahak et al., 2009; de Laat et al., 2010). For example, a previous study with 639 adults with a mean age of 74 years showed that gait velocity was negatively associated with the Fazekas score, an established measure of presence and severity of WMC (Baezner et al., 2008). These results were confirmed in another study (de Laat et al., 2010). A further study in more than 250 older adults with a mean age of 78 years provided evidence that executive functioning, as measured with the Trail Making Test (TMT), is negatively associated with the severity of WMC (Zheng et al., 2012). Another study confirmed these results (Marshall et al., 2006). Also older adults with cognitive impairment showed a negative association of executive functioning with severity and total volume of WMC (Smith et al., 2011).

PD patients show similar associations as older adults without PD. A recent study with 103 early-stage PD patients with a mean age of 65 years showed that executive functioning is negatively associated with total WMC volume (Kandiah et al., 2013). Another study showed that the gait item of the Unified Parkinson's Disease Rating Scale (UPDRS) was positively correlated with the Fazekas score (Sohn and Kim, 1998). Other studies using comparable paradigms and methods confirmed basically these results (Theilmann et al., 2013; Veselý et al., 2015; Veselý and Rektor, 2016).

Overall, there is evidence that presence and severity of WMC influence gait and executive functioning of older adults and PD patients. However, it is, to our best knowledge, not yet investigated whether the factors “age” [used in this study as a surrogate marker for *unspecific* progressive brain degeneration (Fearnley and Leesa, 1991; Moeller et al., 1996; Volkow et al., 1998; Raz, 2000; Smith et al., 2007; Raji et al., 2009)] and “PD” (defined here as a type of *specific* progressive brain degeneration) are associated with distinct WMC-related deficits in gait and executive functioning. Such associations could relevantly affect the design of prospective studies in this field. To test this hypothesis, we investigated and compared WMC, gait and executive functioning in young and old persons with and without PD.

## Subjects and methods

### Participants

This cross-sectional study was performed at the Neurology Department of the University Hospital Tuebingen between 09/2014 and 04/2015. Inclusion criteria were age between 50 and 89 years, the ability to walk at least 20 m with or without walking aid, existence of an axial T2- or FLAIR- (fluid-attenuated inversion recovery) weighted cerebral MRI sequence, and provision of a written informed consent. Exclusion criteria were more than 1 fall per week (for safety reasons), <10 points on the Mini-Mental State Examination (MMSE) (Folstein et al., 1975) (to keep the probability high that the instructions were correctly understood), other neurodegenerative diseases than PD, hospital admission due to acute stroke, WMC obviously due to other causes than microangiopathy (e.g., post-ischemic parenchymal defects >5 cm, vasculitis with cerebral manifestation, hereditary leukoencephalopathy, normal pressure hydrocephalus, multiple sclerosis, infectious and autoimmune encephalitis, brain tumor and metastases). A total of 138 patients who met the above-mentioned criteria were consecutively recruited. Thirty-seven patients were classified as PD, and 101 as persons without PD (Pn). The latter were treated at the neurological ward due to vascular pathologies (transitory ischemic attack, *N* = 10; microangiopathy, *N* = 9), infectious diseases (*N* = 7), epileptic seizures (*N* = 14), pathologies of peripheral nerves (*N* = 23), non-degenerative movement disorders (including functional disorders, *N* = 23), headache/pain (*N* = 9), amnesia (*N* = 4) and orthostatic deficits (*N* = 2). The investigators excluded by interview and clinical observation acute conditions that could relevantly influence the examination. Specifically, any individuals with limiting pain, hemiparesis, disturbed cognition and consciousness were excluded. PD patients were investigated during medication ON phase. Pn and PD subjects were divided in young (yPn, yPD) and old (oPn, oPD) groups using a cut-off of 70 years (Dong et al., 2015). Table 1 shows demographic and clinical parameters of the groups. The study was approved by the ethical committee of the Medical Faculty at the University of Tuebingen. All participants gave written informed consent.

**Table 1:** Demographic and clinical parameters.

	**yPn (1) M (range)**	**yPD (2) M (range)**	**oPn (3) M (range)**	**oPD (4) M (range)**	
Number (female)^*^	65 (49%)	22 (41%)	36 (42%)	15 (27%)	0.4389
Age (years)	57 (50–69)	62 (49–69)	77 (70–89)^1,2^	75 (70–89)^1,2^	**<0.0001**
MMSE [0–30]	29 (15–30)	29 (17–30)	28 (16–30)	28 (17–30)	0.2107
UPDRS III [0–132]	7 (0–38)	41 (16–78)^1,3^	12 (0–46)	38 (15–74)^1,3^	**<0.0001**
BDI II [0–63]	11 (1–51)	9 (5–34)	11 (1–31)	12 (3–20)	0.9894
LED [mg]	–	477 (200–1,350)	–	698 (0–1,500)	0.2046
Anticholinergics [% of total]^*^	2	18^1^	0^2^	13^1,3^	**0.0037**
Cholinergics [% of total]^*^	0	5	0	7	0.1229
Sedatives [% of total]^*^	28	59^1^	19^2^	53^3^	**0.0042**
Fazekas score [0–3]	1 (0–3)	1 (0–2)	1 (0–3)	1 (0–2)	0.0881
TMT-A [s]	36 (20–180)	51 (25–180)	55 (28–180)^1^	68 (36–180)^1^	**<0.0001**
TMT-B [s]	104 (49–300)	254 (53–300)^1^	202 (64–300)^1^	300 (73–300)^1^	**<0.0001**
ST walking [m/s]	0.95 (0.41–1.21)	0.91 (0.69–1.15)	0.87 (0.57–1.26)	0.83 (0.29–1.02)	0.0837
ST subtracting serial 7s [N/s]	0.30 (0.12–0.66)	0.29 (0.14–0.63)	0.22 (0.06–0.93)^1^	0.19 (0.06–0.46)^1,2^	**0.0127**
DT walking while subtracting serial 7s [m/s]	0.82 (0.38–1.14)	0.74 (0.51–1.05)	0.72 (0.44–1.37)	0.69 (0.38–0.86)^1^	**0.0053**
DT subtracting serial 7s while walking [N/s]	0.33 (0.04–0.81)	0.26 (0.10–0.61)	0.31 (0.02–0.59)	0.27 (0.02–0.51)	0.0891
DTC walking while subtracting serial 7s [%]	9.1 (–7.8 to 44.2)	13.1 (–8.5 to 37.1)	13.7 (–19.2 to 37.1)	19.9 (–1.2 to 43.4)	0.0645
DTC subtracting serial 7s while walking [%]	−17.5 (–107.8 to 46.2)	–2.8 (–61.6 to 48.2)	–20.1 (–101.1 to 79.6)	–10.2 (–203.8 to 84.6)	0.4347

Descriptive statistics as well as results of Kruskal-Wallis H test / post hoc Wilcoxon signed-rank tests and

* *Pearson / post hoc Fisher‘s exact test. Significant results (p < 0.05) are in bold*.

1–4 *Significant difference (p < 0.05) compared to the group with the number indicated in the first line; BDI II, Beck Depression Inventory II; DT, dual task; DTC, dual task costs; LED, Levodopa equivalent dosage; M, median; MMSE, Mini-Mental State Examination; N, number; oPD, old patients with Parkinson's disease (PD); oPn, old persons without PD; ST, single task; TMT-A, Trail Making Test part A; TMT-B, Trail Making Test part B; UPDRS, Unified Parkinson's Disease Rating Scale; yPD, young patients with PD; yPn, young persons without PD*.

### Clinical assessment

All study participants underwent an extensive clinical, gait and cognitive assessment, including assessment of medication and medical history taking. Semi-quantitative assessment of motor deficits was performed with the motor part of the revised version of the Unified Parkinson's Disease Rating Scale (MDS-UPDRS III) (Goetz et al., 2008), cognitive assessment with the MMSE (Folstein et al., 1975) and mood assessment with the Beck Depression Inventory II (BDI II) (Beck et al., 1996).

We assessed gait velocity and executive functioning with the following instruments and tasks.

#### Testing simple executive functioning without mobility

The TMT part A (TMT-A) is considered a relatively simple task. It measures visual, motor and processing speed (Bowie and Harvey, 2006). TMT part B (TMT-B) measures cognitive flexibility (Bowie and Harvey, 2006). Both tests are predictors of mobility impairment (Vazzana et al., 2010). They involve the dorsolateral prefrontal cortex, the medial frontal gyrus, the precentral gyrus and the supplementary motor area (Bowie and Harvey, 2006). The TMT has been shown to be sensitive to WMC (Marshall et al., 2006).

#### Testing (simple) mobility

Gait velocity from a 20 m walk at self-selected speed was used as simple mobility task. It reflects physical performance and processing speed (Rosano et al., 2008). The parameter was extracted from the RehaGait® sensor system (Hasomed GmbH, Magdeburg, Germany), a validated gait analysis system (Donath et al., 2016a,b). Based on evidence for the relevance of this parameter (Baezner et al., 2008; de Laat et al., 2010; Hollman et al., 2011), we decided to use gait velocity as the parameter that best reflects quality of gait. The precentral and cerebellar areas, basal ganglia, (Maetzler et al., 2013) fusiform and parahippocampal gyri, bilateral inferior frontal gyri and right supplementary motor area (Jahn et al., 2004) are involved in gait performance.

#### Testing complex executive functioning without mobility

We defined subtracting serial 7s (subtracting a series of 10 steps of 7, starting with a randomly chosen three-digit number) (Hobert et al., 2011) as a complex executive task. Performance of the task requires mathematical skills and attention (Yogev et al., 2008).

#### Testing complex executive functioning combined with mobility

Subtracting serial 7s while walking was used as dual task (DT) paradigm. Participants subtracted serial 7s from a randomly chosen three-digit number while simultaneously walking 20 m in normal pace (Hobert et al., 2011). No instruction for prioritization was given (Yogev-Seligmann et al., 2010). The task requires mathematical skills, working memory and divided attention, (Yogev et al., 2008; Patel and Bhatt, 2014) as well as the activation of supraspinal centers responsible for mobility coordination (Jahn et al., 2004; Maetzler et al., 2013). The following brain areas have been shown to be involved in DT, indicating the complexity of the task: superior (Szameitat et al., 2002; Nebel et al., 2005) and inferior frontal sulcus (Szameitat et al., 2002; Collette et al., 2005; Nebel et al., 2005; Deprez et al., 2013), middle frontal gyrus, (Szameitat et al., 2002; Collette et al., 2005; Nebel et al., 2005; Deprez et al., 2013) superior parietal lobe, (Nebel et al., 2005; Deprez et al., 2013) inferior parietal lobe, (Collette et al., 2005; Nebel et al., 2005) intraparietal sulcus, (Szameitat et al., 2002; Collette et al., 2005; Deprez et al., 2013) anterior cingulate gyrus (Nebel et al., 2005) and cerebellum (Collette et al., 2005; Deprez et al., 2013). Some of these brain areas show a direct and positive correlation with the activity level of the task (Szameitat et al., 2002). Dual task costs (DTC[%] = (ST-DT)/ST^*^100) were defined as the relative change in performance during DT compared with single task (ST). Positive DTC reflect a decrease in performance of DT compared with ST.

#### Testing coordination and prioritization aspects, and complex executive functioning combined with mobility

“Coordination and prioritization during DT” can be considered a very complex task with involvement of coordination, prioritization and mobility (Hobert et al., 2011; Buss et al., 2014). DTC may best define quality of this performance. Besides the brain areas already activated for the performance of DT, an activation of the following brain areas has been associated with coordination and prioritization: bilateral middle frontal gyri, left posterior inferior frontal sulcus, right anterior superior frontal gyrus and prefrontal cortex (Szameitat et al., 2016).

### Assessment of white matter changes

**Figure 1:**
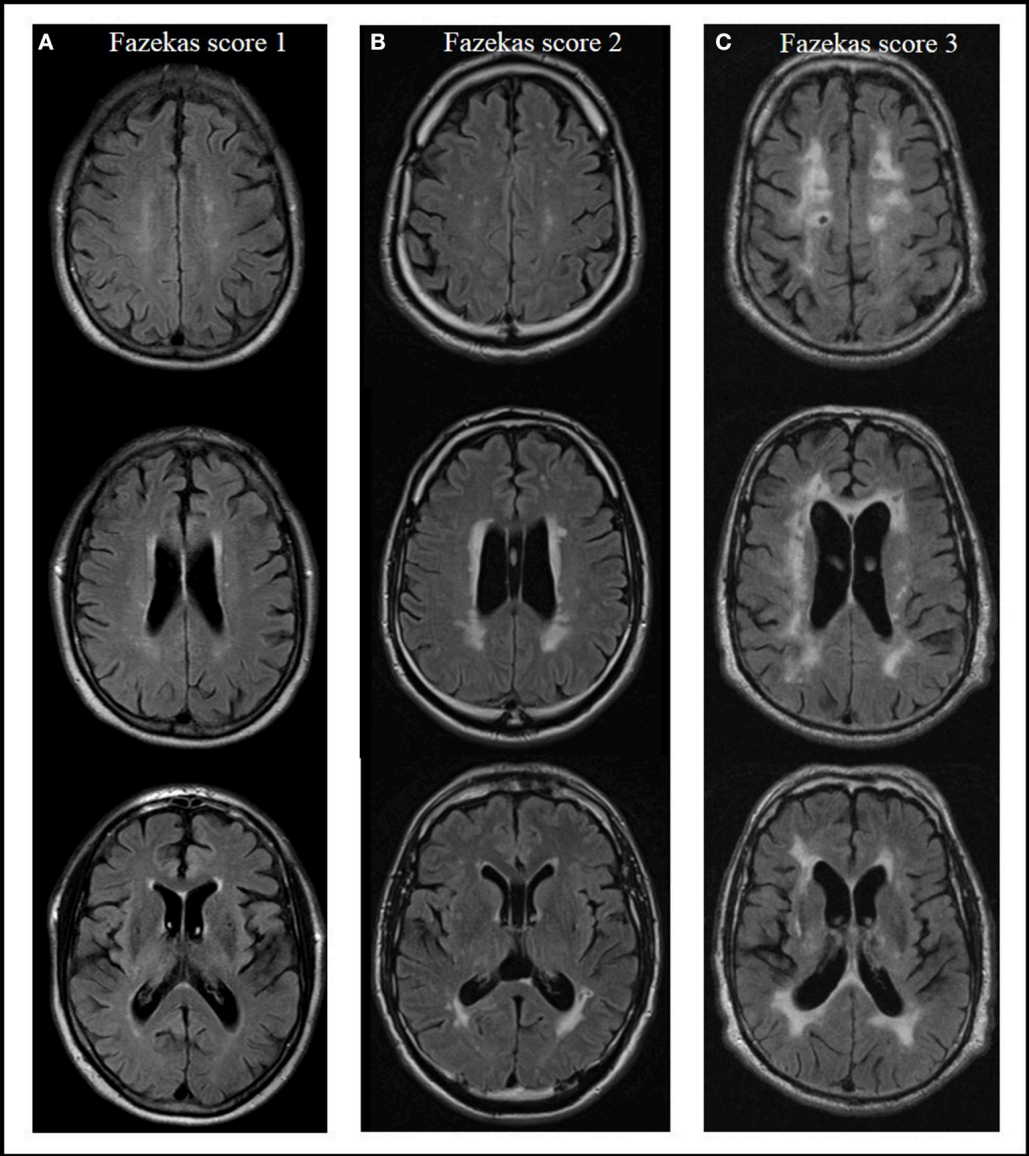
Presence and severity of white matter changes in MRI according to the Fazekas score, examples from the actual evaluation. Score 1 (mild) with focal or punctuate lesions **(A)**; score 2 (moderate) with beginning confluent lesions **(B)**; score 3 (severe) with confluent lesions **(C)** (Prins and Scheltens, 2015).

Severity of WMC was rated on axial T2- or FLAIR-weighted images from routine clinical MRIs using the Fazekas score (Fazekas et al., 1987) in a modified version according to Prins and Scheltens (2015), which is well established for defining different groups by severity of WMC (Van Straaten et al., 2006; Ropele et al., 2009). Absence of lesions in subcortical and periventricular regions was rated with a score of 0. Focal or punctuate lesions (diameter of single lesions < 10 mm, diameter of grouped lesions <20 mm) were rated with a 1 (mild); beginning confluent lesions (diameter of single lesions between 10 and 20 mm, diameter of grouped lesions more than 20 mm, with no more than connecting bridges between individual lesions) were rated with a 2 (moderate); and confluent lesions (single lesions or confluent areas of more than 20 mm) with a 3 (severe) (Prins and Scheltens, 2015). Rating of lesions was done by a single rater blinded to group status. Figure 1 shows examples of different WMC severity scores.

## Statistics

Demographic, clinical, WMC and gait and executive function parameters across the four groups are presented with median and range, or frequency. Statistical analysis was performed with Kruskal-Wallis H / *post-hoc* Wilcoxon signed-rank test, or Pearson / *post-hoc* Fisher's exact test. We calculated Spearman correlations between WMC and the gait and executive performances for each group. For comparisons of these correlations between groups, we performed Fisher's z-transformations. This method accounts for the respective Spearman's rho and sample size of each of two groups. Data were analyzed using the statistical software program JMP® version 11.2.0 (SAS Institute Inc.).

## Results

Table 1 provides details about demographic, clinical, medication, WMC, gait and executive function parameters of the groups. The yPn and yPD, as well as oPn and oPD were not relevantly different regarding demographic and clinical scores, respectively. As expected, both groups without PD performed better in the UPDRS than the PD groups (*p* < 0.001). Levodopa equivalent dose was not significantly different between yPD and oPD, and the proportion of participants using cholinergic medication was comparable across groups (*p* = 0.12). The proportion of participants using sedatives (*p* = 0.004) and anticholinergics (*p* = 0.004) was significantly different across groups, with comparably low proportions of study participants without PD that took these drugs. Presence and severity of WMC were comparable between the groups (Supplementary Table). Regarding gait and executive function parameters, yPn performed better in TMT-A, TMT-B and ST subtracting serial 7s compared to oPn and oPD, better in DT walking while subtracting serial 7 compared to oPD, and better compared to yPD in TMT-B. yPD performed better than oPD in ST subtracting serial 7s.

**Figure 2:**
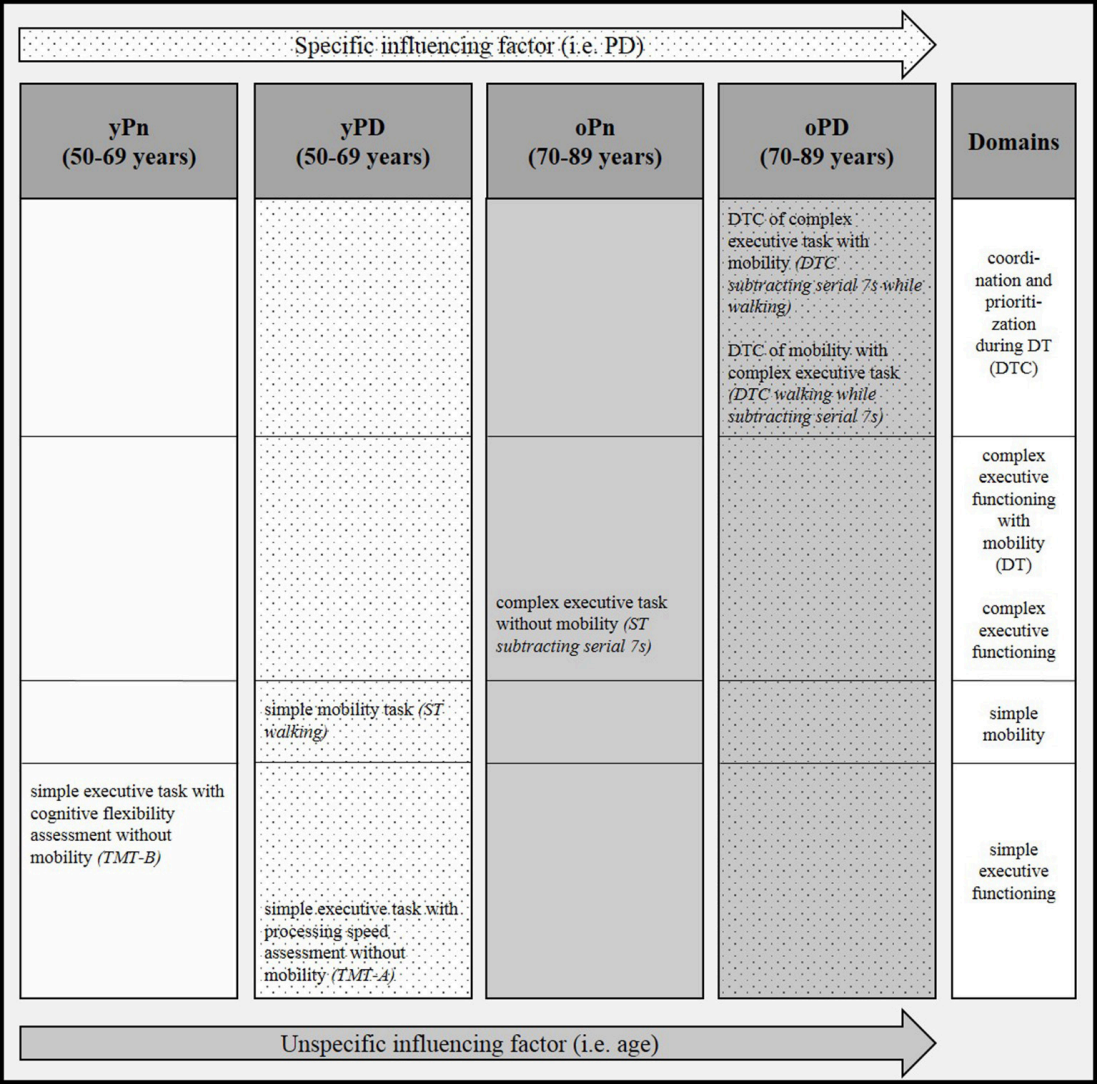
Pattern of significant correlations of severity of white matter changes (WMC) with gait and executive function parameters across four groups with and without advanced age and Parkinson's disease (PD). DT, dual task; DTC, dual task costs; oPD, old patients with Parkinson's disease (PD); oPn, old persons without PD; ST, single task; TMT-A, Trail Making Test part A; TMT-B, Trail Making Test part B; yPD, young patients with PD; yPn, young persons without PD.

Details of the correlation analyses between the Fazekas score and respective gait and executive function parameters are presented in Table 2 and Figure 2. The following correlations were significant:

- In yPn, the Fazekas score correlated with the TMT-B. This value was not significantly different from the values of the other groups, respectively.
- In yPD, the Fazekas score correlated with the TMT-A. This correlation was significantly different from the oPn and the oPD values. yPD also showed a correlation of the Fazekas score with ST walking. This value was significantly different from the result found in yPn.
- In oPn, the Fazekas score correlated with the ST subtracting serial 7s value. This result differed significantly from the yPn values.
- In oPD, the Fazekas score correlated with both DTC parameters, i.e., walking while subtracting serial 7s (significantly different compared to yPn and oPn values) and subtracting serial 7s while walking (significantly different compared to the yPn value).

**Table 2:** Correlation coefficients between white matter changes, and gait and executive function parameters, sorted by groups.

	**Spearman's ρ yPn (1)**	**Spearman's ρ yPD (2)**	**Spearman's ρ oPn (3)**	**Spearman's ρ oPD (4)**
N	65	22	36	15
TMT-A [s]	0.19	0.58^**^^,^^3,4^	0.07	−0.48
TMT-B [s]	0.33^*^	0.37	0.29	−0.11
ST walking [m/s]	0.05	−0.61^**^^,^^1^	−0.31	−0.11
ST subtracting serial 7s [N/s]	0.03	−0.19	−0.40^*^^,^^1^	0.19
DT walking while subtracting serial 7s [m/s]	−0.05	−0.02	−0.20	0.27
DT subtracting serial 7s while walking [N/s]	0.08	−0.37	−0.22	0.39
DTC walking while subtracting serial 7s [%]	0.14	−0.32	0.02	−0.63^*^^,^^1,3^
DTC subtracting serial 7s while walking [%]	0.01	0.05	−0.33	−0.56^*^^,^^1^

*White matter changes were rated with Fazekas score. Correlation coefficients were calculated with Spearman's rank correlation*.

* p < 0.05;

** *p < 0.01*.

*Significant (p < 0.05) group differences in correlations between groups are indicated in bold and by the group number (^1–4^)*.

*DT, dual task; DTC, dual task costs; N, number; oPD, old patients with Parkinson's disease (PD); oPn, old persons without PD; ST, single task; TMT-A, Trail Making Test part A; TMT-B, Trail Making Test part B; yPD, young patients with PD; yPn, young persons without PD*.

## Discussion

This study confirms the already known influence of WMC on gait and executive functioning (Schmidt et al., 1993; Baezner et al., 2008; Blahak et al., 2009; de Laat et al., 2010; De Laat et al., 2011; Zheng et al., 2012; Theilmann et al., 2013; Prins and Scheltens, 2015). This effect has been shown to be, at least partly, driven by cognitive reserve (Scarmeas et al., 2003; Steffener et al., 2009; Brickman et al., 2011; Tucker and Stern, 2011; Stern, 2012; Stern et al., 2012; Fernandez-Cabello et al., 2016). Moreover, our results argue for a distinct and selective association between “specific” (here defined by PD pathology) and “unspecific” (age-related) brain pathologies and WMC-related gait and executive function deficits. This latter aspect cannot be explained by cognitive reserve models and requires further investigation. Surprisingly, groups with no or only mild (age or PD-related) signs for brain pathology (additional to WMC) reflected these associations only in relatively simple tests, and the groups with signs for relevant (age and PD-related) brain pathology only in relatively complex tests. This effect is in our view not explained by potential confounders, such as medication, as groups with relatively high proportions of participants taking, e.g., anticholinergics and sedatives, did not show comparable results regarding the association between WMC-related executive dysfunction and age and PD.

The group suggested having no or minimal unspecific and specific brain pathology (yPn) was the only one that showed a significant association of WMC with TMT-B performance. The TMT-B is a relatively simple test of cognitive flexibility (Bowie and Harvey, 2006) and involves circumscribed brain areas (Bowie and Harvey, 2006). The group suggested having no or minimal unspecific and some specific brain pathology (yPD) showed significant associations of WMC with TMT-A performance and with ST walking. All three above-mentioned parameters (TMT-A, TMT-B, walking) can be considered as highly processing speed-dependent (Bowie and Harvey, 2006; Rosano et al., 2008), again with limited and circumscribed involvement of brain areas (Jahn et al., 2004; Bowie and Harvey, 2006; Maetzler et al., 2013). In fact, deficits in processing speed-dependent tasks have already been shown in young PD patients (Lee et al., 2003). Based on our results it is tempting to hypothesize that WMC in middle-aged brains that have no or only mild or specific (here: dopaminergic) pathology affect mainly and relatively selectively processing-speed dependent tasks. In other words, processing speed dependent tasks are obviously tasks that are receptive to age-related brain changes at this stage of life (50–70 years of age). This stage may provide the best target and target range for WMC that affect areas such as the dorsolateral prefrontal cortex, the medial frontal gyrus, and the precentral gyrus (Bowie and Harvey, 2006; Smith et al., 2011).

The significant association of WMC with ST walking performance in PD patients may further support this hypothesis: We included patients with Hoehn and Yahr stage 1–3, i.e., with relatively preserved gait but already existing walking deficits (Mancini et al., 2009; King et al., 2012). An additional pathology, such as WMC, may target here a function that is in a vulnerable stage without relevant compensation capacity [omitting a ceiling effect (Maetzler and Hausdorff, 2012)] and not as advanced to provoke floor effects. In fact, we could not find a significant association between WMC and ST walking performance in oPn and oPD, and floor effects may at least partly explain the lack of significant association of WMC with some other test parameters (e.g., TMT-B values in oPD, Table 2).

Attention-related tasks might best reflect the association between age and WMC. The oPn group showed a significant association of WMC with ST subtracting serial 7s, a task relevantly influenced by attention (Yogev et al., 2008). Comparable to the constellation described in the above paragraph for less affected groups and other parameters, the largest range of values of ST subtracting 7s (0.06–0.93, Table 1) in this cohort suggests that this task provides a good target and target range for adults with advanced age.

The group defined as having age- and PD-related brain pathology (oPD) showed significant correlations of WMC with DTC, which we consider a surrogate for a very complex task involving many brain areas (Szameitat et al., 2016), i.e., the simultaneous performance of coordination and prioritization, and complex executive functioning combined with mobility. Interestingly, these correlations were negative, indicating that more WMC lead to lower DTC. The only reasonable explanation for this phenomenon is, in our view, an unexpectedly poor ST performance. Patients with multiple brain pathologies may regularly suffer from apathy, depressive symptoms and loss of motivation, which may be less prevalent under challenging conditions (Metzger et al., 2016). Another reason may be that these simultaneously performed actions trigger each other reciprocally by serving as internal pulse generators for the respective other task. This phenomenon has been demonstrated in 10 healthy subjects with a mean age of 32 years, who showed an interference of gait rhythm with fast finger-tapping under dual tasking conditions (Ebersbach et al., 1995). The posture second theory as proposed by Bloem et al. (2006) can explain only parts of the observed effect and may not relevantly contribute to the explanation of the specific issue described here.

A limitation of the study is that we recruited the cohort from the ward of a neurological department. We thus cannot exclude that in particular the yPn and oPn groups do not exactly reflect the general population. Moreover, group sizes were relatively unbalanced. This aspect may also explain why many but not all significant correlation values within groups were also significantly different to all other groups, respectively. We still feel that our results are valid and relevant, as the stable pattern of investigated significant associations between WMC and executive function deficits across the four groups is suggestive of a certain principle driving these results. We were also able to extensively evaluate the general and neurological performances and to use additional assessment information (such as brain imaging) to ensure that no obvious additional pathology was present. Still, a re-evaluation of the findings presented here with larger group sizes, a population-based control group and potentially longitudinal design would certainly strengthen our results. Such a study could also test association aspects between age and PD as well as medication interactions, which were not specifically addressed here. Furthermore, consideration of cortical atrophy could also be an interesting addition to such analyses. As a general comment, the observed effects should be interpreted in the context of the expected median differences in test performances across the groups (see also Table 1). Groups suggested having no or mild brain pathology showed overall better executive functioning and gait levels than those suggested having specific and unspecific brain pathology. It is also noteworthy that general cognition and mood do obviously not relevantly drive our results: The MMSE and BDI II values were similar across groups.

In conclusion, age and PD-related brain pathology seem to interact with WMC-related gait and executive function deficits. Our results should be taken into account when assessment batteries for studies investigating the influence of WMC on gait and executive functioning in older adults and patients with neurodegenerative diseases are designed. When confirmed in future studies, these results may also have an influence on pragmatic management of older adults with WMC and neurodegenerative diseases in combination with executive and gait deficits.

## Author contributions

JS, KB, FB, MAH, and WM made substantial contributions to the acquisition, analysis and interpretation of data for the work. JS, KB, and FB contributed equally to this study. JG was also responsible for data analysis. JS, MAH, and WM drafted the paper. MH and TG made substantial contributions in technical implementation, data acquisition and analysis of the data. Mt, MB, TL, and PS made substantial contributions in supervision of the MRI analysis. MC and SH made substantial contribution to the revision including data generation and statistical expertise. All authors gave their final approval of the version to be published and agree to be accountable for all aspects of the work in ensuring that questions related to the accuracy or integrity of any part of the work are appropriately investigated and resolved.

### Conflict of interest statement

The authors declare that the research was conducted in the absence of any commercial or financial relationships that could be construed as a potential conflict of interest.
