# Corrigendum: White Matter Changes-Related Gait and Executive Function Deficits: Associations with Age and Parkinson's Disease

**DOI:** 10.3389/fnagi.2017.00342

**Published:** 2017-10-17

**Authors:** Jennifer Sartor, Kristina Bettecken, Felix P. Bernhard, Marc Hofmann, Till Gladow, Tobias Lindig, Meltem Ciliz, Mara ten Kate, Johanna Geritz, Sebastian Heinzel, Marije Benedictus, Philip Scheltens, Markus A. Hobert, Walter Maetzler

**Affiliations:** ^1^Department of Neurodegeneration, Center for Neurology and Hertie Institute for Clinical Brain Research, University of Tuebingen, Tuebingen, Germany; ^2^German Center for Neurodegenerative Diseases (DZNE), Tuebingen, Germany; ^3^Hasomed GmbH, Magdeburg, Germany; ^4^Department of Diagnostic and Interventional Neuroradiology, University of Tuebingen, Tuebingen, Germany; ^5^Alzheimer Center and Department of Neurology, Neuroscience Campus Amsterdam, VU University Medical Center, Amsterdam, Netherlands; ^6^Department of Neurology, Christian-Albrechts-University of Kiel, Kiel, Germany

**Keywords:** cognition, dual tasking, older adults, Parkinson's disease, white matter changes

In the published article, there was an error regarding the affiliation for Jennifer Sartor. As well as having Affiliation 1 and 2 she also contributed equally.

The corrected affiliation appears above.

The authors apologize for this error and state that this does not change the scientific conclusions of the article in any way.

The original article has been updated.

## Conflict of interest statement

The authors declare that the research was conducted in the absence of any commercial or financial relationships that could be construed as a potential conflict of interest.

