# Supplementary material for: White Matter Changes-Related Gait and Executive Function Deficits: Associations with Age and Parkinson's Disease
Source: Front Aging Neurosci. 2017 Jun 30;9:213. doi: 10.3389/fnagi.2017.00213 (PMC5491602; doi:10.3389/fnagi.2017.00213)
Supplement: Supplementary file 1 [file DataSheet1.docx]

**Supplementary Table.** Presence and severity of white matter changes, according to the Fazekas scale, separated by groups

|  | **yPn** | | **yPD** | | **oPn** | | **oPD** | |
| --- | --- | --- | --- | --- | --- | --- | --- | --- |
|  | N | Frequency [%] | N | Frequency [%] | N | Frequency [%] | N | Frequency [%] |
| Score 0 | 26 | 40 | 6 | 28 | 7 | 19 | 3 | 20 |
| Score 1 | 35 | 54 | 15 | 68 | 24 | 67 | 10 | 67 |
| Score 2 | 3 | 5 | 1 | 5 | 3 | 8 | 2 | 13 |
| Score 3 | 1 | 1 | 0 | 0 | 2 | 6 | 0 | 0 |
| Total | 65 | 100 | 22 | 100 | 36 | 100 | 15 | 100 |

Frequency, proportion of participants of the respective group. Results are rounded. N, number; oPn, old persons without Parkinson’s disease (PD); oPD, old patients with PD; yPn, young persons without PD; yPD, young patients with PD
